# Valvulotomy of the great saphenous vein in ex situ non-reversed and in situ setting: a multicenter post-market study to assess the safety and efficacy of the AndraValvulotome™”

**DOI:** 10.1007/s00423-023-03189-5

**Published:** 2023-11-30

**Authors:** Yaser Souri, Dominik Liebetrau, Alexander Hyhlik-Dürr, Vascular Investigators, E. Weigang, J. Kemke, D. Branzan, M. Wilhelmi, S. Classen, K. Pfister, M. Kuhnert, S. Seifert, W. Derwich, K. Stavroulakis

**Affiliations:** Vascular and Endovascular Surgery, Medical Faculty, University of Augsburg, Stenglinstrasse 2, 86156 Augsburg, Germany

**Keywords:** Valvulotomy, Bypass surgery, Non-reversed bypass, Infrainguinal bypass, Great saphenous vein bypass

## Abstract

**Purpose:**

To evaluate the safety and technical success of the AndraValvulotome™ device (Andramed GmbH, Reutlingen, Germany) in patients with peripheral arterial disease (PAD) requiring bypass surgery using the great saphenous vein (GSV) as graft.

**Methods:**

This was a multicenter, post-market observational study conducted in 2021 in 11 German centers. Safety and efficacy data were prospectively collected and analyzed. Primary endpoints were the absence of device-related serious adverse events until 30 ± 7 days follow-up, the clinical efficacy of valvulotomy, which was defined as pulsatile blood flow in the bypass and the number of insufficiently destroyed vein valves. Secondary endpoints were the number of valvulotomy passages, the primary patency rate of the venous bypass (determined by a color-duplex sonography showing a normal blood flow through the bypass and absence of stenosis or occlusion), and the primary technical success defined as the absence of product-specific (serious) adverse events and clinical efficacy.

**Results:**

Fifty-nine patients were enrolled. The mean age of the patients was 71 years (46–91), and 74.6% were males. The vein material used for bypass grafting had a median length of 47.5 cm (range 20–70 cm) with a median diameter of 5.0 mm (range 3–6 mm) and 4.0 mm (range 2–6 mm) in the proximal and distal segments, respectively. The technical success rate was 96.6%. The primary patency rate was 89.9% at 30 days follow-up. The clinical efficacy was rated as very good in 81% of patients, fair in 17%, and poor in 2%. Between 1 and 5 (average 2.9) valvulotome passages were performed. One product-related serious adverse event was recorded (bypass vein dissection).

**Conclusion:**

The AndraValvulotome™ can be considered a safe and effective device to disrupt venous valves during in situ non-reversed bypass surgeries using GSV grafts in patients with PAD.

## Introduction

The first-line treatment of symptomatic peripheral arterial disease (PAD) is often endovascular. Percutaneous transluminal angioplasty is a minimally invasive treatment that has been established in the last decades and is showing significantly better results due to improved technical skills and technological advancement in devices and equipment used. However, the problems of restenosis and early and mid-term vessel occlusions have not yet been completely eliminated despite technological advances such as the introduction of drug-coated balloons and stents or vessel preparation techniques such as atherectomy [1]. The results of endovascular treatment are still unsatisfactory, especially in long and highly calcified lesions [2]. Recent data support the benefit of bypass surgery in these cases [2]. Especially when the great saphenous vein (GSV) is available, bypass surgery should be the first line treatment due to its good mid- and long-term results with high evidence recommendation [2–4]. In below-knee (BK) bypass surgery, in situ or non-reversed technique using GSV is one of the well-established and standardized procedures [5]. Complete valve disruption using valvulotomy is one of the key points to achieving adequate bypass flow and, therefore, long-term durability. One of the most common reasons for early graft failure is inadequate disruption of valves during in situ bypass surgery [6, 7]. The use of fixed-size or adjustable valvulotomes showed no significant difference in efficacy, whereas an adjustable valvulotome may be more beneficial [6, 7].

There is a need for more data regarding this topic, as insufficiently disrupted valves still pose a common problem during vein bypass surgery which can negatively influence this approach [6, 7]. Safety, efficacy, as well as effectiveness, are reported in a small number of reports and different settings (minimally invasive, Hydro-Valvulotome), mostly during in situ bypass surgeries [8–10]. Evidence concerning safety and efficacy during valvulotomy, including the AndraValvulotome™, in bypass surgery is still lacking and must be improved.

Application of an active post-market surveillance observation study design is not only a part of market drug analysis but also an integral part of research in medical devices to help and ensure a safe and efficient use [11].

Therefore, the purpose of this observational study was to evaluate the safety and efficacy of the AndraValvulotome™ (Andramed GmbH, Reutlingen Germany) in a multicenter setting in patients with severe PAD.

## Materials and methods

**Fig. 1:**
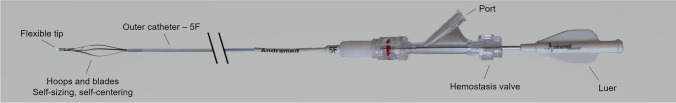
Overview of the AndraValvulotome™

This multicenter, prospective, post-market observational study was planned to assess the safety and efficacy of the AndraValvulotome™ (Fig. 1) in patients with symptomatic severe PAD who were scheduled for peripheral bypass surgery using the great saphenous vein (GSV) as a bypass graft. All patients gave their written informed consent to participate in this study. The local Ethics Committee (Ref. 21–0172) of each participating center approved the study and its protocol. A total of 11 sites (5 university hospitals and 6 community hospitals) enrolled patients between April 2021 and May 2022. The study was registered on DRKS (DRK0025919) and Clinical Trials (NCT04815473).

A total of 62 patients were screened***.*** Patients who suffered from PAD Rutherford stage ≥ 3 with a minimum length of the GSV of 20 cm were included in the study. All patients requiring bypass surgery using GSV who met all inclusion/exclusion criteria were included in this study. Patients with varicose veins as well as patients with life expectancy of less than 1 year were excluded. The bypass procedure was performed according to the standard of care of each participating institution, and the surgical technique was chosen according to the preference of the surgeon. The surgeon estimated the pulsatile blood flow in the bypass clinically, classifying it as very good, fair, and poor. Antiplatelet and anticoagulation regime was implemented according to each hospital protocol.

To assess the suitability of the vein for bypass surgery and to quantify the valves needing to be disrupted, color-coded duplex sonography was used. After the valvulotomy, color-coded duplex sonography or digital subtraction angiography was performed to determine the insufficiently disrupted valves.

Patients were followed up clinically and with duplex ultrasound at 30 ± 7 days after the primary procedure. The following parameters were recorded: antiplatelet and anticoagulation regime, ankle-brachial index (ABI), changes in the Rutherford stage, bypass patency, additional procedures, and adverse events.

Primary endpoints of the study were the absence of device-related serious adverse events up to 30 ± 7 days during follow-up, the clinical efficacy of the valvulotome based on the presence of pulsatile blood flow in the bypass after valvulotomy, and the rate of inadequately disrupted venous valves. A device-related serious adverse event is an event that is causally related to the investigational product leading to death, permanent impairment of a body function, prolongation of hospital stay, or the need of medical or surgical procedure to prevent life-threatening illness or injury or permanent impairment of a bodily function. Secondary endpoints were the number of valvulotomy passages, the primary patency rate of the venous bypass (determined by a color-duplex sonography showing a normal blood flow through the bypass and absence of stenosis or occlusion), and the primary technical success defined as the absence of product-specific (serious) adverse events and clinical efficacy.

## Statistical analysis

Patient data, including demographics, preoperative risk factors, and intraoperative and postoperative outcomes, were recorded in an electronic database. Data were analyzed using the statistical software R (R 4.1.2 (2021–11-01), The R Foundation for Statistical Computing, c/o Institute for Statistics and Mathematics, Wirtschaftsuniversität Wien, Vienna, Austria). Categorical variables are presented as number (percentages), and continuous variables are presented as mean or median (range). To compare the categorical variables of the Intention-To-Treat (ITT) cohort to the comparative study of Malmstedt et al. (2005) [11], Fischer’s exact tests were used. The cumulative primary patency rate was derived using the Life Time (LT) method of Rutherford et al. (1997).

## Results

**Fig. 2:**
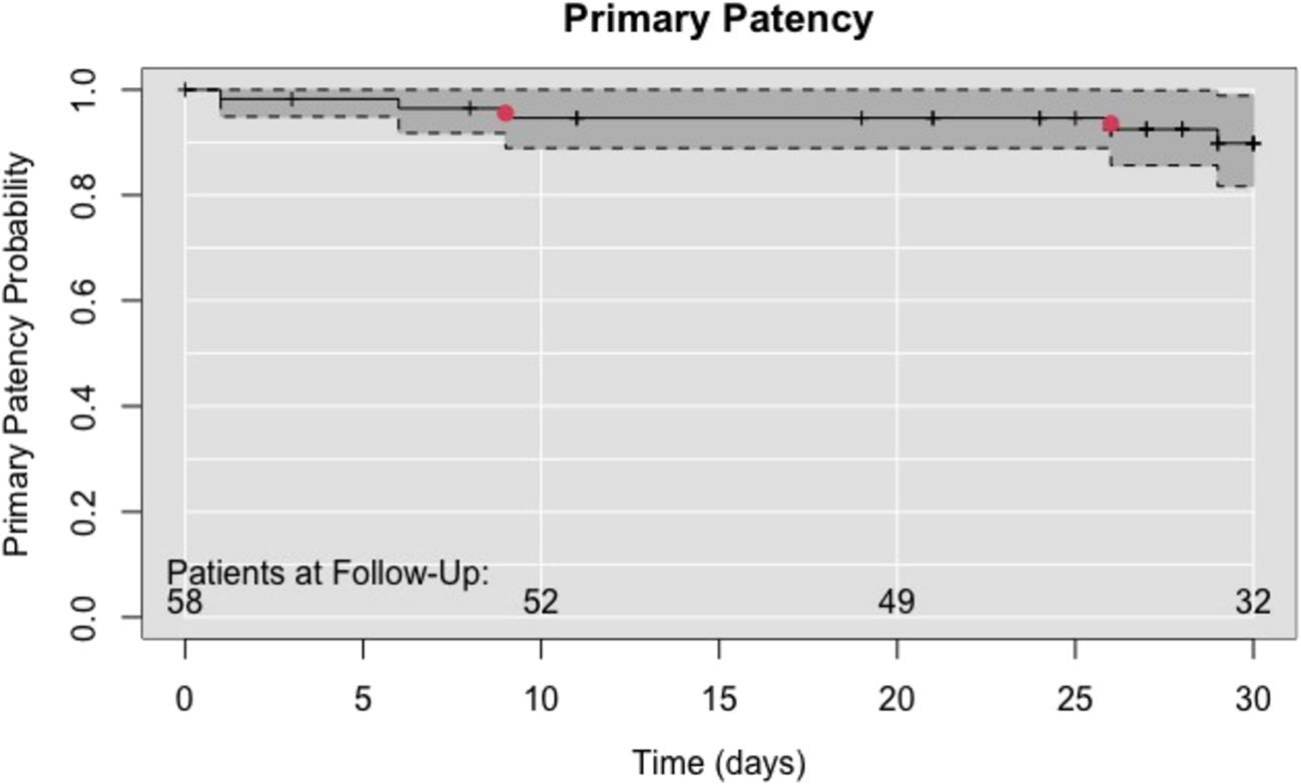
Kaplan–Meier plot. The number of patients planned for follow-up is shown in the figure with an interval of 10 days. A total of 58 patients were treated and planned for follow-up. Red dots indicate the moment of complete bypass occlusion. At every step down, an additional intervention was performed to keep the bypass open which decreases the primary patency rate. A vertical bar indicates the moment when a patient was seen last or came to follow-up. The primary patency rate reached 89.9%

Fifty-nine patients with a median age of 71 years (range: 46–91 years) with severe symptomatic PAD were included in the study. Forty-four (74.6%) of those were males. Patients’ demographics and indications for treatment are summarized in Table 1. Most patients underwent treatment for peripheral arterial disease (PAD) at Rutherford stages 5 (49%) and 3 (33%). Rutherford stage 6 was observed in just 5% of the patient population. The AndraValvulotome™ was used in 58 evaluable patients. Due to miscommunication, one patient was enrolled in the study but was treated using a different valvulotome. A total of 266 venous valves of the GSV were determined on pre-operative ultrasound, and 260 valves were successfully disrupted using the valvulotome. Only 6 valves were not successfully disrupted, equivalent to a success rate of 98%. Two of those valves were located in the proximal part of the GSV, one in the mid portion, and three in the distal part of the vein. The smallest and largest diameters of the vein were 2.0 mm and 6.0 mm, respectively. The length of the veins varied between 20 and 70 cm. The GSV as a bypass graft was implanted in a non-reversed fashion in all patients and left in situ in 62.1% of patients. The distal bypass anastomosis was performed in 44.8% of the patients at the level of the popliteal artery. Adjunctive intraoperative procedures such as minor amputation or thromboendarterectomy (TEA) were performed in 22 patients. The primary technical success rate was 96.6% achieving a pulsatile arterial blood flow in the bypass with the absence of device-related adverse events. Table 2 shows the characteristics of the veins, the location of the distal anastomosis, and the primary patency rate. The primary patency rate reached 89.9% after 30 ± 7 days due to two complete bypass occlusions and three reinterventions to keep the vein graft patent. The two bypass occlusions were noticed 9 days and 26 days after index procedure. For both occlusions, no causal factors were described. For the reinterventions, two percutaneous transluminal angioplasties (PTA) with stent implantation on the distal part of the bypass were performed. In one case was the site of intervention not documented. Figure 2 illustrates the primary patency rate in a Kaplan–Meier plot.

**Table 1:** Patient demographics and comorbidities

	Patients *n* = 59	Patients %
Patient characteristics		
Age, year	71 (46–91)	
Men	44	74.6
Comorbidities		
Pre-operative ABI median (range)^b^	0.44 (0.0–1.4)	
Hypertension	52	88.1
CAD	26	44.1
COPD	7	11.9
Stroke	7	11.9
Diabetes	24	40.7
Smoking (≤ 10 years) and current	50	84.7
BMI (kg/m^2^) mean (range)	26.12 (17.76–48.83)	
Renal insufficiency^c^	12	20.3
Hyperlipidemia	33	55.9
Vascular surgery	44	74.6
Rutherford classification (0–6)		
1	0	0
2	0	0
3	18	33
4	9	15
5	29	49
6	3	5

*ASA*, American Society of Anesthesiologists; *BMI,* body mass index; *CAD*, coronary artery disease; *COPD*, chronic obstructive pulmonary disease

^a^Continuous data are presented as the median (range); categorical data are given as the counts (percentage)

^b^*ABI*, Ankle-brachial index; based on 52 values (= 7 missing, 3 of them due to medial sclerosis), 1 of the 52 entries with value “0.7–1.0” set to 0.85, 1 of the 52 entries with value “ > 1.4” set to 1.4

^c^Creatinine > 2.0 mg/dL

**Table 2:** Vein mapping, patency

Distal anastomosis		
Popliteal artery	26	44.8%
Tibio-peroneal trunk	9	15.5%
Peroneal artery	6	10.3%
Tibialis posterior artery	7	12.1%
Tibialis anterior artery	5	8.6%
Dorsalis pedis artery	1	1.7%
Others*	4	6.9%
Vein characteristics	Median	Range
Length	47.5 cm	20.0–70.0 cm
Diameter		
Proximal	5.0 mm	3.0–6.0 mm
Distal	4.0 mm	2.0–6.0 mm
Smallest segment	3.35 mm	2.0–6.0 mm
Patency		
Primary (53/58)	53	89.9%
Bypass occlusions	2	3.4%
Primary reinterventions (< 30 days) ^a b c^	3	5.2%

^a^Renew distal vein segment

^b^PTA distal anastomosis popliteal artery

^c^PTA distal anastomosis crural

^*^Jump grafts to foot arteries in combination with popliteal or crural distal anastomosis

The clinical efficacy based on distal pulsatile blood flow was rated as very good in 81%, fair in 17%, and poor in 2%. Between one and five valvulotomy passages were performed to achieve satisfactory results (mean 2.9).

The rate of device-related intraoperative bleeding was 0%. A total of 22 adverse events were recorded; out of these was one device-related serious adverse event, where a dissection of the GSV was noticed intraoperatively resolved with an interposition using a Dacron graft.

### Clinical outcomes

Patients that came for bypass surgery had a Rutherford stage between 3 and 6. After the surgery, an improvement was noticed at the time of discharge and follow-up for the majority of patients as it is shown in Table 3.

**Table 3:** Rutherford classification by patients (preoperative, discharge, and follow-up)

	0	1	2	3	4	5	6
PreOP	0	0	0	18	8	29	3
Discharge	19	7	9	1	0	20	0
Follow-up	7	13	13	1	1	13	2

As shown in Table 4, there was a significant improvement in ABI after bypass revascularization. The ABI mean increased significantly with *p* < 0.001 for both PreOP vs. discharge and PreOP vs. follow-up (Table 4).

**Table 4:** Ankle-brachial index by patients (preoperative, discharge, and follow-up)

	Min	25%	50%	75%	Max	Mean
PreOP	0.00	0.31	0.45	0.60	1.40	0.54
Discharge	0.33	0.61	0.82	1.00	1.22	0.80
Follow-up	0.24	0.73	0.91	1.00	1.77	0.87

No major amputations were performed in any of the patients, whereas 9 (15.5%) of the patients required minor amputations.

## Discussion

This multicenter study demonstrates the high safety and efficacy of venous valve disruption of the GSV during in situ and non-reversed bypass using the AndraValvulotome™ in patients with severe PAD. This presents the first set of data for the AndraValvulotome™ in this indication and patient population.

Current recommendations indicate that bypass surgery should be used as a first-line modality of treatment in patients with PAD if adequate GSV is present [2]. Published data showed that adverse limb events or death from any cause is significantly lower in bypass surgery compared to endovascular treatment (42.6% vs. 57.4%, *p* < 0.001) [2]. Superior efficacy of bypass surgery compared to endovascular treatment was also demonstrated with lower rates of above-ankle amputation, myocardial infarction, and stroke. The length of hospital stay was the only parameter favoring endovascular treatment for patients with chronic limb-threatening ischemia [2]. With this in mind, it is important to have safe and effective instruments like valvulotomes, to be able to perform bypass surgeries with long-term patency in a multitude of patients and anatomies.

The AndraValvulotome™ Post-Market Study was designed based on the study of Malmstedt et al. (2005) [8]. Malmstedt et al. recruited 30 patients, but only 14 patients with 61 inspected valves were treated with one similar device, where the disruption of the valves was assessed by using angioscopy. In contrast to this study with 58 patients and 266 valves, we examined almost twice as many patients and four times as many valves.

In our analysis, clinical efficacy was determined by assessing blood flow after usage of the AndraValvulotome™ in a subjective assessment. A total of 98% of the blood flow was defined as very good or fair. Only in 1 case (2%), blood flow was declared as poor, which was caused by 3 remaining valves distally in the vein graft. In Malmstedt et al. (2005), the results for the blood flow with objective assessment via angioscopy are similar, except Malmstedt did not have any flow which was assessed as poor, although not in all cases the valves were completely disrupted [8]. The assessment of the blood flow in our study was performed subjectively which could have had an impact on the outcome; however, the results can be interpreted as good.

During the study, three primary endpoints were analyzed. The primary safety endpoint, the absence of device-related serious adverse events (SAE), was analyzed. Only one clearly device-related SAE (dissection of the vein) was documented. Furthermore, one possible device-related SAE (formation of hematoma) and one unknown device-related SAE (local wound-healing disorder) were reported out of a total of 22 adverse events. Hence, a complete absence of device-related events was not achieved throughout the study. However, all defined SAEs, which had a certain relationship to the device, can occur since they are described as possible complications in the instructions for use (IFU) when using a valvulotome. Also, in the study of Troisi et al. (2019), device-related injuries to the vein were reported, but the severity was not categorized [9]. This shows that also with the valvulotome used in other studies, device-related adverse events can occur. Therefore, it is crucial that surgeons have experience with the device being used and bail out strategies available.

A total of 266 valves were evaluated; only six valves (2.26%) in four vein grafts (6.9%) after valvulotomy with the AndraValvulotome™ failed to be disrupted. Compared with Malmstedt et al. (2005), the AndraValvulotome™ shows a very high efficacy regarding disrupting venous valves. Malmstedt detected 26.2% incompletely disrupted valves of 61 inspected valves in total when using a comparable valvulotome. Furthermore, the quantity of grafts with remained valves in this study corresponds to a similar result from Gangadharan et al. with remained valves in 3 of 37 cases (8.1%) [10].

For the secondary endpoints, two secondary safety endpoints were analyzed: first, the frequency and severity of device-related bleeding over the duration of study participation which did not occur in any patients; second, the frequency and severity of device-related SAEs and AEs, where 1 SAE was reported to be clearly related to the device and defined as severe. In the study of Troisi et al. (2019), in 6 cases vein injury (vein adventitial damage) were reported due to the in situ technique with the LeMaitre valvulotome®, and in 1 case bleeding was detected [9]. Therefore, the results regarding device-related events during the AndraValvulotome™ Post-Market Study are acceptable.

Other secondary endpoints, including the number of valvulotome passages, the primary bypass patency rate, and the primary technical success were analyzed. During the valvulotomy, a mean of 2.9 passages were performed within a range between 1 and 5 passages. These results correspond to the study of Troisi et al. (2019), where an average of 2.6 passages with a range of 1–5 passages [9].

The primary patency rate after the index procedure was 100%, which fell to 89.9% at follow-up (Fig. 2). The primary patency, with the expandable LeMaitre valvulotome® in the Malmstedt study at 30 days follow-up, was 100%. Several factors could impact the outcome on the primary patency, including stage severity of the PAD, comorbidities, or experience of the vascular surgeon in using valvulotome.

Primary technical success, which was defined as pulsatile blood flow with the absence of device-related adverse events, was 96.6% after the index procedure. In the study of Troisi et al. (2019), the technical success rate was 100% [9]. However, the definition of technical success in that study was a patent bypass with pulsation after the usage of the LeMaitre valvulotome®. Therefore, the result of the technical success rate with the AndraValvulotome™ can also be rated as good.

In conclusion, the AndraValvulotome™ can be considered a safe and effective device to disrupt venous valves during in situ non-reversed bypass surgeries using GSV grafts in patients with PAD.

## Limitations of the study

The study was performed as a multicenter trial in 11 centers, and the interpretation of the findings was carried out by different surgeons. Therefore, the results were subjected to bias. An example can be clearly seen in the assessment of blood flow in the bypass according to the quality of pulsation in the bypass, which could have been evaluated more objectively using other methods such as angioscopic techniques. These are however difficult to implement in everyday clinical practice since they are not available in every clinic [12]. Another way to overcome this obstacle and minimize bias in future studies is by using core labs to evaluate the findings.

Although only 58 patients were treated with the AndraValvulotome™ in the study, a high number of 266 valves were analyzed for disruption, which seems to be sufficient. Patients with other diseases that also require a venous bypass, e.g., popliteal aneurysm, can be included in future studies, as well.

Additionally, this study is a single-arm study. A direct comparison with a competitive device was not an objective, and the impact of patient characteristics on outcomes could not be analyzed. Furthermore, a better evaluation of the study results can be achieved with a longer follow-up of the patients.
